# Springtime precipitation effects on the abundance of fluorescent biological aerosol particles and HULIS in Beijing

**DOI:** 10.1038/srep29618

**Published:** 2016-07-29

**Authors:** Siyao Yue, Hong Ren, Songyun Fan, Yele Sun, Zifa Wang, Pingqing Fu

**Affiliations:** 1 State Key Laboratory of Atmospheric Boundary Layer Physics and Atmospheric Chemistry, Institute of Atmospheric Physics, Chinese Academy of Sciences, Beijing 100029, China; 2 College of Earth Sciences, University of Chinese Academy of Sciences, Beijing 100049, China; 3 Center for Excellence in Urban Atmospheric Environment, Institute of Urban Environment, Chinese Academy of Sciences, Xiamen 361021, China

## Abstract

Bioaerosols and humic-like substances (HULIS) are important components of atmospheric aerosols, which can affect regional climate by acting as cloud condensation nuclei and some of which can damage human health. Up to date, release of bioaerosols and HULIS initiated by precipitation is still poorly understood. Here we present different release processes for bioaerosols, non-bioaerosols and HULIS during a precipitation event in Beijing, China. Large fungal-spore-like aerosols were emitted at the onset and later weak stage of precipitation, the number concentration of which increased by more than two folds, while the number concentration of bacteria-like particles doubled when the precipitation strengthened. Besides, a good correlation between protein-like substances that were measured simultaneously by on-line and off-line fluorescence techniques consolidated their applications to measure bioaerosols. Furthermore, our EEM results suggest that the relative contribution of water-soluble HULIS to microbial materials was enhanced gradually by the rain event.

Primary biological aerosol particles (PBAP)^1^,^2^, such as pollen, fungal spores, bacteria, viruses, proteins and fragments of organisms, and humic-like substances (HULIS)^3^,^4^,^5^ are ubiquitous components in atmospheric aerosols. They have been proposed to have significant influence on regional weather, climate and air quality through many processes^6^,^7^,^8^,^9^,^10^,^11^,^12^,^13^. For example, many studies have revealed that PBAP can act as cloud condensation nuclei (CCN) and ice nuclei (IN) efficiently in warm conditions compared with inorganic compounds^3^,^12^,^14^,^15^,^16^,^17^,^18^. Besides, Vaïtilingom *et al*.^19^ suggested the role of microorganisms in metabolizing organic compositions by reducing the source of radicals in the atmosphere. Mortazavi *et al*.^20^ discussed potential effects of microorganisms on the melting and processing activities of snowpack. Some HULIS were found to be effective surface-active species^21^, thus they could possibly serve as CCN and influence the microphysics of clouds^22^,^23^. In addition, PBAP and HULIS have been discovered to exert adverse effects on human health^24^,^25^,^26^. For instance, Fang *et al*.^27^ reported high increase of *Alternaria*, both plant pathogen and human allergen, from April to May in urban Beijing. Dou *et al*.^28^ revealed that the production of reactive oxygen species in human cells could be mediated by HULIS. However, because of the complexity in the composition, emission and evolution of atmospheric aerosols, these effects are still poorly understood^6^,^7^,^8^.

Among many processes, precipitation has already been found to correlate with the release of PBAP^29^,^30^,^31^. Raindrop splash of fungal spores have been reported since the early 1950s^29^,^32^,^33^,^34^. Huffman *et al*.^31^ observed intensifications of bacteria and fungal spores during and after precipitation and discovered two previously unknown fungi species, *Acremonium implicatum* and *Isaria farinosa*, which could serve as IN. However, detailed observation and quantification for the release of PBAP initiated by precipitation are sparse, which is important for understanding of rain-forced shift on atmospheric aerosol compositions and on modeling of the land-atmosphere interaction^35^, especially in urban regions. Moreover, studies of the precipitation effect on HULIS abundance are still inadequate.

In this study, from a field observation of a spring rainfall event in Beijing, time-resolved characteristics of fluorescent biological aerosol particles (FBAP) and non-FBAP were described quantitatively by a Wideband Integrated Bioaerosol Sensor (WIBS-4, Droplet Measurement Technologies, Inc.)^36^,^37^. Fluorescent aerosols detected by WIBS could be regarded as a lower estimate of PBAP^10^,^31^,^38^. The temporal variations of fluorescence properties of water-soluble organic matter (WSOM) in 3-hour aerosol samples were measured by an off-line excitation-emission matrix (EEM) fluorescence spectrophotometer (FluoroMax-4), which were compared to the results of WIBS. Besides, humic-like and protein-like substances determined by EEMs gave further insights into the variations of their abundance and relative contributions to water-soluble materials.

## Results

### Rain event

**Figure 1:**
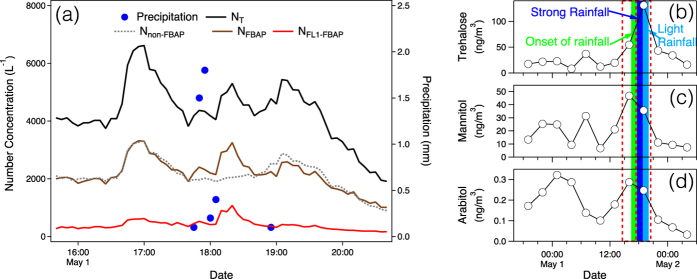
Temporal variations of aerosol number concentration (>0.8 μm, optically scattering diameter) observed by WIBS and rainfall rate and organic marker compounds. (**a**) Variables presented are number concentration of total particles (N_T_), fluorescent biological aerosol particles (N_FBAP_), channel 1 fluorescent biological aerosol particles (N_FL1-FBAP_ , protein-like aerosols) and non-fluorescent biological aerosol particles (N_non-FBAP_). Five-minute accumulated rainfall rate is shown by blue dots. Organic molecular markers, that is, (**b**) trehalose, (**c**) mannitol and (**d**) arabitol, are given along with the precipitation phases (colored shades, defined in Fig. 5). The red dotted boxes show the time periods of the offline samples.

The sampling site was surrounded by a public park covered mostly with pine trees, a yard with vegetation and a small river on its north side. It was influenced by air flows from west and north, where the park and the river are located, respectively (Figure S1). The rain event lasted around one hour during May 1, 2014 (Fig. 1). Considering that the detection limit for the rainfall was 0.1 mm, the rain event was perceived to start at around 16:40 and end before 20:00 based on the change of WIBS results. For this small-scale rain event, it was rational to regard the surrounding soil, vegetation and water body as the main source of released aerosols^39^. The whole measurement period was separated into three sessions, before, during and after rain. Five-minute accumulated rainfall rate is show in Fig. 1. The rain was weak at the beginning, then it enhanced to approximately 1.8 mm (5-minute accumulated). Finally, the precipitation diminished gradually.

### OC, WSOC and WSTN concentrations

The mean mass concentration of organic carbon (OC) before the rain event was 16.8 μgC m^−3^ (Table 1). The result was in consistent with a previous study^40^. During the precipitation, it surged to 39.7 μgC m^−3^, approximately by a factor of two. After the rainfall, it decreased to the level of 15.8 μgC m^−3^ similar to that before the light shower. Similar trends were observed for water-soluble organic carbon (WSOC) and water-insoluble organic carbon (WIOC), which increased to 9.0 μgC m^−3^ and 30.7 μgC m^−3^ during the precipitation, respectively. Primary biological materials containing proteins are one of the sources for organic nitrogen. During the precipitation, the increase of water-soluble total nitrogen (WSTN) was also observed. As reported before, PBAP^2^,^41^ and HULIS^42^ could be significant sources of atmospheric OC. Thus, the increased WSOC and WIOC mass could possibly result from the enhancements of PBAP and HULIS.

### Organic tracers

In Fig. 1b, arabitol and mannitol, two tracer compounds of fungal spores, increased at the earlier stage of the precipitation. The mass concentration of trehalose, which has various sources such as microorganisms and fugitive soil dust, also increased in the precipitation event. However, the concentration peak occurred during its latter period.

### Fluorescence of WSOC

**Figure 2:**
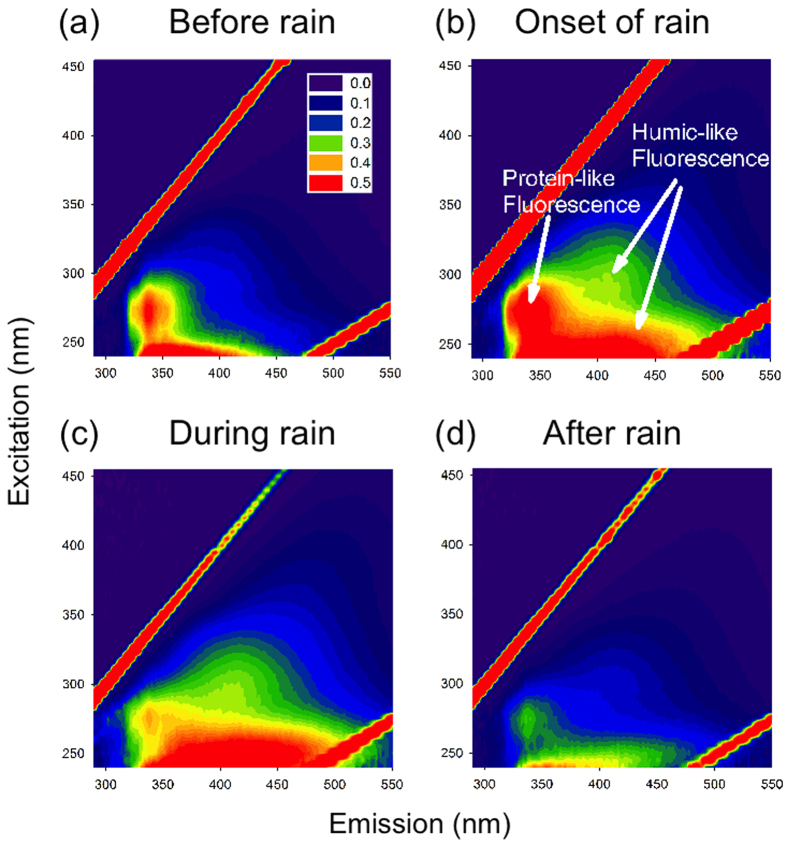
Typical fluorescence spectra (EEM) of water-soluble organic carbon (WSOC) for selected filter samples (in RU L m^−3^). They are resolved from samples in periods (**a**) before rain: 08:26–11:22 (May 1), (**b**) onset of rain: 14:24–17:22 (May 1), (**c**) during rain: 17:24–20:21 (May 1) and (**d**) after rain: 02:22–5:20 (May 2).

The trends of fluorescent water-soluble components can be displayed by EEMs (Fig. 2). The intensities of the fluorophores were calibrated to Raman unit followed by the air volume of each sample in order to make a comparison among samples^43^. The intensity of protein-like substances (Peak T) increased at the onset of the rainfall, however, it decreased in the latter stage. Meanwhile, the quantity of Peak A (humic-like substances) showed a continuous increase during the shower. Finally, both peak T and peak A decreased after the rainfall. For further exploration, complete time series of EEMs are given in the Supplement materials (Figure S4), covering periods before, during and after the precipitation.

### FBAP

**Figure 3:**
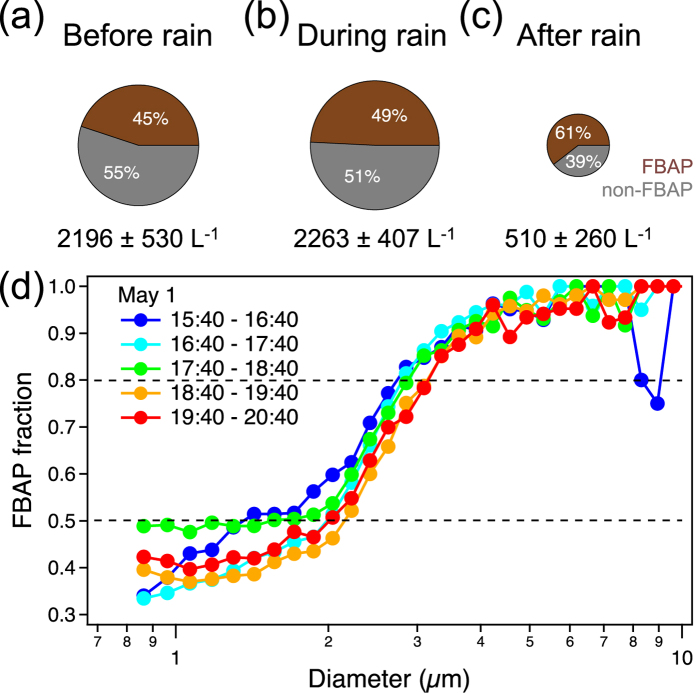
FBAP fractions in total aerosol particles for three periods and size-resolved FBAP fraction in five intervals around rainfall. (**a**) Before rain: 17:30 (April 30)–11:30 (May 1), (**b**) during rain: 14:30–20:30 (May 1) and (**c**) after rain: 20:30 (May 1)–17:30 (May 2). The averages (also represented by the circle areas) and standard deviations of the number concentrations of FBAP in these periods are also presented. (**d**) Size distributions of FBAP fraction for five consecutive periods.

Figure 3 presents number concentrations of FBAP and their fractions in total particles in three periods: before, during and after the rain event. Although the abundance of FBAP decreased after the rainfall (down to 510 L^−1^), their ratios increased from 45% to 49% and then to 61%. From a size-resolved fraction analysis (Fig. 3d), nearly 50% of the particles larger than around 2 μm are of biological origins. For aerosols larger than 3 μm, only less than about 20% of them are non-FBAP.

Figure 1 shows number concentrations of total, fluorescent and non-fluorescent biological aerosols, which allows a detailed examination of their temporal variations. Extended time series are provided in the Supplementary material (Figure S5). During the rain event, three intermittent peaks were observed. In the first period, the enhancement of particles was the most prominent, with contributions from both FBAP and non-FBAP. During the second period, while rainfall was in its highest strength, the loadings of non-FBAP did not increase as much as that of FBAP and its previous interval. For the last peak span, both FBAP and non-FBAP increased with weaker intensity compared with that in the first interval.

## Discussion

The temporal variations of organic and nitrogen components (Table 1) revealed an increase during the precipitation followed by a decrease after the rainfall for both WSOC, WIOC and WSTN. Such a pattern was consistent with the variations of number concentrations of fluorescent biological particles and non-fluorescent biological aerosols (Fig. 1). Mechanisms of these intensifications can be attributed to several sources, such as generation of particles by raindrop impact on soil, water bodies and fungal spores released from leaf surfaces^31^,^44^,^45^. As reported earlier, OC contains a considerable proportion of biological materials and humic-like substances, which comprise both water-soluble compositions and water-insoluble substances^2^,^41^,^42^.

**Figure 4:**
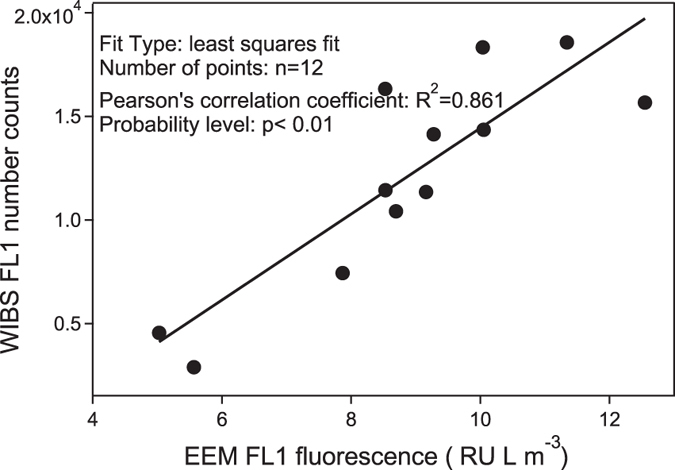
Linear correlation between number counts of WIBS FL1 channel and EEM intensity summed in corresponding excitation-emission zone. The EEM total fluorescence intensities were calculated in the range of Excitation/Emission: 275–285 nm/330–400 nm.

**Figure 5:**
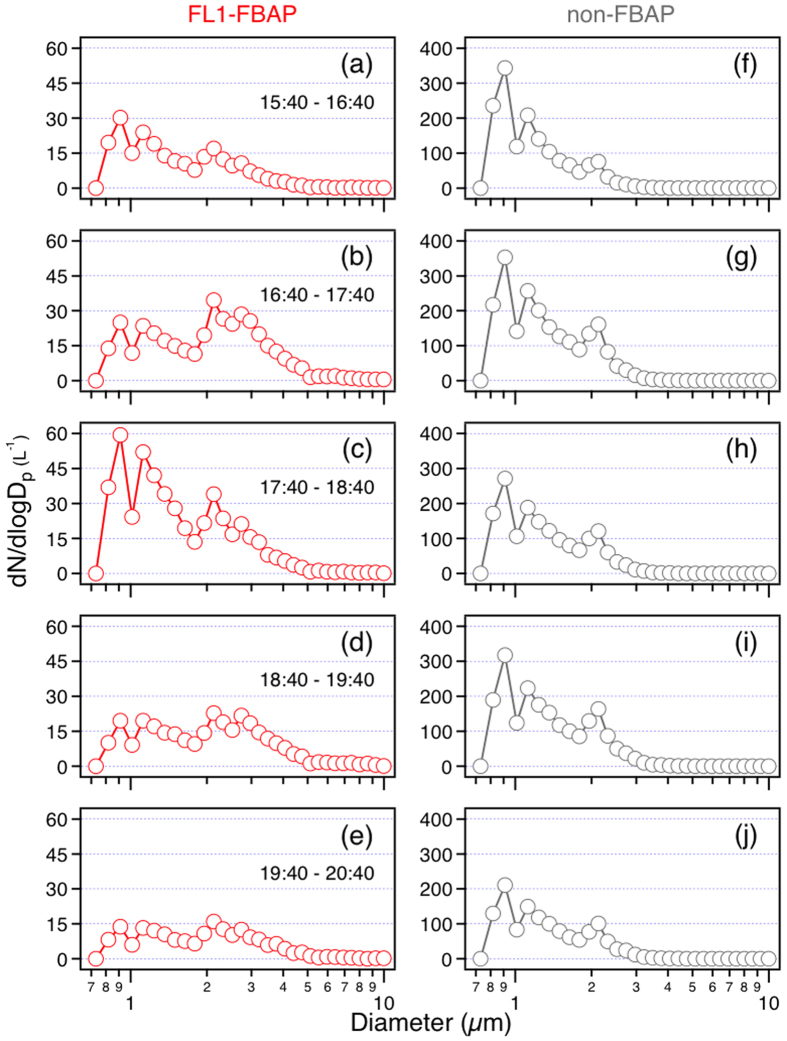
Size distributions of FL1-FBAP and non-FBAP in five consecutive periods for the rain event (May 1, 2014). Graphs in the same row are in the same periods.

The increasing trend of FBAP fraction (Fig. 3) indicates an enhanced release or a low removal rate of biological materials compared to non-biological species. To better understand these processes, high time-resolution measurements of PBAP were performed by WIBS. As the FL1 fluorescence channel of WIBS correlated well with the off-line EEM measurement of protein-like substances (Fig. 4), this channel was perceived with high credibility to represent biological aerosols containing proteins. Hence, FL1-FBAP was selected to compare with non-FBAP to reveal their contrast characteristics along this precipitation (Fig. 5). The varying trends of the characteristics of FL2-FBAP and FL3-FBAP were similar to FL1-FBAP. Quantitative data for non-FBAP and FBAP in all three fluorescence channels were provided in the supplement (Table S1).

In the first stage, the increase of both FL1-FBAP and non-FBAP in the size modes of 2.2 μm and 2.8 μm were up to two folds and three folds, respectively. However, the surge of FL1-FBAP for particles larger than 3 μm was nearly by four times, which is higher than that of non-FBAP (less than 3 folds). This suggests a more significant release of biological particles, which were very probably bacteria and fungal spores (2–3 μm), lichens, animal dander and plant fragments (>3 μm) mechanically agitated from surfaces^29^,^33^,^41^. The increasing leaf wetness as a possible factor for bioparticle emission was reported by Butterworth *et al*.^46^ and other studies (e.g., Huffman *et al*.^31^). The increase of non-FBAP in this phase was most likely due to raindrop splashes on soil, leaves and water surface^45^. These FBAP (e.g. *Drechslera turcica*) and non-FBAP could also be previously attached to leaf surfaces or soil land by static electricity and then be repelled due to the change of relative humidity^33^,^47^. In addition, in this phase, the number concentrations of non-FBAP increased in all size modes, reflecting a physical cause which was omnipresent for particles varying wide size span.

However, in the second phase, as the rainfall strengthened, large FL1-FBAP (>2.2 μm) and all non-FBAP were suppressed by rain scavenging. In contrast, the abundance of fine FL1-FBAP (size modes 1.2 μm and 0.9 μm) increased by a factor of two. The decrease of fine non-FBAP could be attributed to rain scavenging. Considering that this mechanism could also be applied to biological particles, therefore, the active release of these biological aerosols should be much stronger. These particulate matters were probably bacteria, most of which being small, intact organisms^1^.

As the precipitation abated in the third phase, both the increasing trends and the increasing quantities of non-FBAP of all size modes were similar to that in the first stage. This added support to the dynamic emission mechanism for non-fluorescent biological particles. However, the feature for FL1-FBAP differed from that in the first period. The number concentration of FL1-FBAP larger than 3 μm still increased slightly, which indicates that fungal spores continue to emit at a minor level during the late period of the rain event. But, the number concentration of FL1-FBAP in 2.2 μm size mode decreased, contrary to that in the first phase. This feature distinguished them from those of sizes larger than 2.8 μm. Therefore, our results suggest that different bioaerosols exist in different sizes; their emission behaviours differ during the rain event. As to particles smaller than 1.2 μm, their abundance once again decreased as in the first period. Hence, the onset of rainfall could be advantageous for their removal from the atmosphere, and intense precipitation could be beneficial for their release. Finally, as the rain diminished, dry deposition and the weakness of boundary layer turbulent transport caused the decrease of the total particle levels.

Combining the offline organic tracer measurements, new insights could be provided about the physical properties of the particles observed by WIBS. Considering the observed augmentation of the fungal spore tracers, arabitol and mannitol^48^,^49^,^50^, at the onset of the rainfall, the 2–3 μm size mode particles could be perceived as fungal spore particles (Fig. 1b). As to trehalose, this compound comes from microorganisms like fungal spores, bacteria and fugitive soil dust^50^,^51^,^52^,^53^. The peak value of trehalose was in the ninth sample. However, the number concentrations of non-FBAP particles, for both total amount (Fig. 1) and size-segregated categories (Fig. 5), reached their peaks at the earlier stage of the rainfall, occurring in the eighth sample. Besides, the mass concentration of fungal spore particles was also highest for the eighth filter. Thus, it is suggested the increased particles in the 1.2 μm size mode were smaller bacteria.

**Figure 6:**
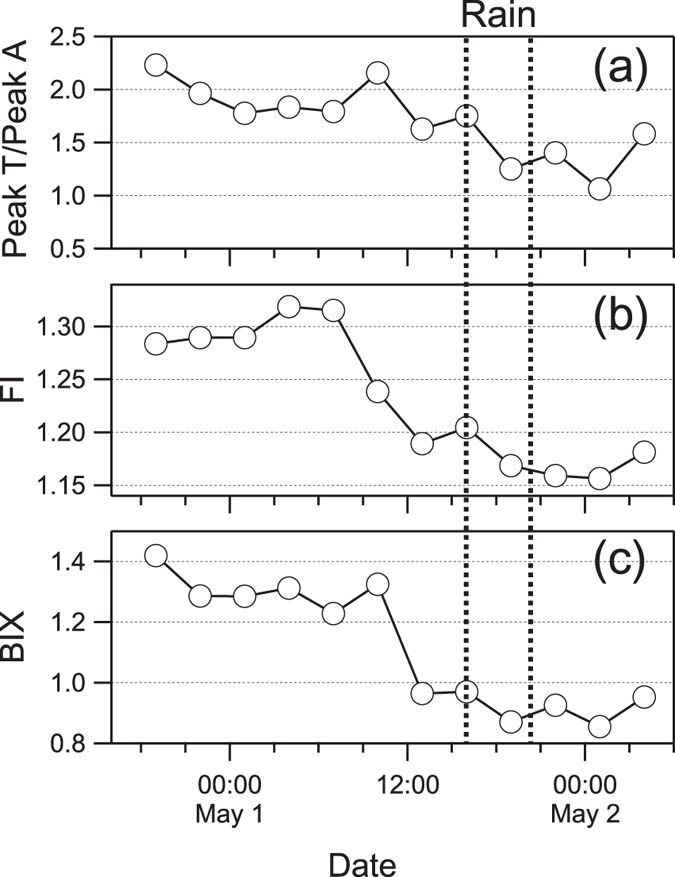
Temporal variations of fluorescence indices of WSOC. (**a**) The relative abundance of protein-like fluorescence (Peak T) to humic-like fluorescence (Peak A). (**b**) Fluorescence index (FI) and (**c**) biological index (BIX) are indices representing relative contribution materials of terrestrial and biological sources.

As to water-soluble HULIS, EEM results showed an increase in particles during precipitation, and a decreased loading after the rain event, which was less than that before the rain event (Fig. 2). For the sake of a semi-quantitative description of the relative contribution of terrestrial and microbial sources to organic matter, two indicators, the fluorescence index (FI) and biological index (BIX) were calculated^54^,^55^,^56^. FI is an indicator reflecting the relative proportion of dissolved organic matter (DOM) derived from microbial and terrestrial sources. A value larger than 1.9 indicates a mainly microbial source and low aromaticity, whereas a value smaller than 1.4 suggests large proportions of terrestrial substances and high aromatic carbon amount^43^. BIX is a proxy for an assessment on the contribution of autochthonous biological activity to DOM^57^. Values larger than 1 reflects a significant microbial source apportion or fresh biological organic matter, while values no larger than 0.6 implicates scarce microbial materials. The time series of FI and BIX are shown in Fig. 6. At the onset of the precipitation, all three indices increased suggesting an intensified contribution of microbial materials to the water soluble contents of aerosols. However, the overall decrease of FI, BIX and Peak T/Peak A ratio implies an increasing relative contribution of terrestrial non-biological substances for the water-soluble contents of total aerosols. This agrees well with previous analysis of EEM results, supporting the conclusion that substantial aerosols were generated from soil, which were very possibly emitted by impaction of raindrops on land^45^.

Conclusively, we observed different release and removal mechanisms of biological and non-biological particles responding to different rainfall conditions and enrichment of water-soluble HULIS compared to bacterial substances due to precipitation. FBAP in five size modes differ each other on their liberation from the surfaces. Although, after the rainfall, the number concentration of all particles decreased, the dispersion of biological materials and abiotic substances are still important for issues such as biodiversity and nutrients distributing. Thus, for description and modeling of rain affected land-atmosphere interaction, these mechanisms should be considered separately. In addition, the enrichment of HULIS compared to bacterial substances in water-soluble fractions of atmospheric aerosols was observed during the rain event in urban Beijing. Considering the widespread and high frequencies of rainfall around the world^58^, and their multiple influence on health, atmospheric oxidative ability, surfactant characteristics as CCN and radiation effect on local and global climate^3^,^6^, it is important to investigate the rain effect on biological aerosols and HULIS enrichment in different geographical locations in future studies.

## Methods

### Site and meteorological data

Collecting of aerosol samples and the performance of WIBS were conducted on the rooftop of the Hexi building located at the State Key Laboratory of Atmospheric Boundary Layer Physics and Atmospheric Chemistry (LAPC), Institute of Atmospheric Physics (IAP), Chinese Academy of Sciences in Beijing, China. The site is situated to the east of the Yuan Dadu Walls Relic Site Park, which is covered by conifer pine trees. The land is covered by sparse grass mulch with bare surface soil exposed. There is a small river to the north of the site. Meteorological data were measured at the level of 8 m above ground that is nearly as high as the inlet of WIBS and filter sampling. Besides, rainfall data were collected by a rain gauge (a tipping bucket, TEM525, Texas Electronics, Inc.) located at the ground meteorological station. Precipitation was recorded every two seconds, and the lower limit of detecting was 0.1 mm. The rainfall provided were cumulated over five minutes. Besides, all time are presented in local time format.

### Filter Sampling

Ambient total suspended particles (TSP) were sampled onto quartz fibre filters by a gravimetric volume sampler (Zambelli, Italy) for around 3 hours for each (from 18:00 on April 30 to 18:00 on May 2, 2014). Two blank samples were collected with the pump off during the sampling. The filters were previously enveloped with aluminum foils and then baked at 450 °C for 6 hours. Sampled filters were immediately stored in a refrigerator under −20 °C until the analysis. Methods for analysis of the quartz filters were the same as Fu *et al*.^43^.

### OC, WSOC, WSTN and offline fluorescence analysis

Organic carbon and elemental carbon were analysed by Semi-Continuous OC-EC Field Analyser (RT3205, Sunset Laboratory Inc.). Prior to the measurement of samples, 10 μL sucrose solution (42 μgC μL^−1^) was used to calibrate the baseline for three times. An aliquot (14 mm diameter) of each quartz filter was cut for analysis of water soluble substances. To analyse water-soluble materials, the samples were extracted by 20 mL of Milli-Q water under ultra-sonication for 15 minutes. Then, the water extracted was filtrated by a syringe-driven filter (Millex-GV, 0.22 μm) and analysed by TOC analyser (TOC-L, Shimadzu) for WSOC and WSTN. WIOC was calculated as the subtraction of WSOC from OC. And the mean values of blanks were abstracted from all ambient samples.

To determine the fluorescence characteristics of water soluble materials, a fluorometer (Fluoromax-4, Horiba) and a UV-Vis spectrophotometer (U3900H, Hitachi) were used for analysis. For sake of comparison, EEMs were corrected by the fluorescence intensity and the volume of sampling for the area of the aliquot and the amount of water used for extraction^43^. EEMs contain valuable information about the compositions of water-soluble parts of the aerosols. According to Coble^59^, the zones of an EEM can be categorized into peaks of specific types of substances. Typical peaks observed in these samples were Peak T (excitation/emission: 275–280 nm/330–340 nm, protein-like), Peak A (290–310 nm/370–430 nm, humic-like) and Peak M (260 nm/400–460 nm, humic-like). For each peak value, they were calculated by summarising the nine fluorescent intensities around the maximum value found in the region. As Peak M could be found in samples of both marine and terrestrial sources^3^, Peak A was selected here as an indicator of humic-like substances. In addition, fluorescence index (FI)^55^ and biological index (BIX)^56^ were used as indices of relative contributions of biological materials and humic-like substances to water-soluble contents of aerosols. FI is calculated by dividing fluorescence intensity at 370 nm/450 nm (excitation/emission) by the value at 370 nm/500 nm. BIX is the ratio of 310 nm/380 nm to 310 nm/430 nm.

### Organic Molecular Tracers Measurement

The method used to extract and analyse organic compounds is same to that reported previously^48^,^50^. A filter aliquot was extracted with dichloromethane/methanol (2:1, v/v) for three times, under ultrasonication for 10 minutes. The extracted solution was filtered through silica wool. Then, it was concentrated using a rotary evaporator and then was nitrogen-stripped to dryness. Derivatization was then performed by reaction with 30 μL of N,O-bis-(trimethylsilyl)trifluoroacetamide (BSTFA), consisting of 1% trimethylsilyl chloride and 10 μL of pyridine at 70 °C for 3 hours. After that, 30 μL of *n*-hexane containing 1.43 ng μL^−1^ of the internal standard (C_13_ *n*-alkane) were added into the derivatives prior to gas chromatography/mass spectroscopy (GC-MS) analysis. GC-MS analyses were performed on an Agilent model 7890 GC coupled to Agilent model 5975 C MSD. Separation was realized on a DB-5MS fused silica capillary column (30 m × 0.25 mm i.d., 0.25 μm film thickness) with a GC oven temperature program. The GC injector was kept at 280 °C. The MS part was operated on Electron Ionization (EI) mode at 70 eV and scanned over the range span of 50–650 Da. The response factors for target compounds were determined with authentic standards. Field blank membrane was analysed through the same procedure and was subtracted from the real samples. Recoveries of sugars were better than 80%.

### WIBS

The Wideband Integrated Bioaerosol Sensor is an instrument that utilizes fluorescence to detect bioaerosols, together with information about the optical scattering diameter of the particles and their morphology. Each inhaled particle is firstly illuminated by a 635 nm diode laser to determine its scattering size based on Mie theory. The refractive index assumed by Mie theory here is established on aerosols of standard mono-disperse polystyrene latex (PSL) microsphere. Simultaneously, the forward-scattered light is collected by a multi-element forward-scattering detector to determine the particle shape. As the particle falls down, two xenon flash-tubes centred on 280 nm (tuned to detect tryptophan molecule) and 370 nm (tuned to detect biofluorophore, NADH) are initiated if the particle size exceeds a predefined threshold. And the fluorescent emissions are collected and split to two fluorescence detection bands, ranging 310–400 nm and 420–650 nm, separately. And because the elastically scattered 370 nm excitation saturated the first emission detection band the fluorescence information for each aerosol are reflected by three channels, namely FL1 (280 nm excitation ∼310–400 nm emission), FL2 (280 nm excitation ∼420–650 nm emission), FL3 (370 nm excitation ∼420–650 nm emission).

The WIBS-4 version can be performed either in high gain (targeted to measure particles between 0.5 μm to 12 μm) or in low gain (targeted to measure particles between 3 μm to 31 μm)^60^. In this study, the high gain setting was chosen in order to explore smaller particles. Besides, as reported, the counting efficiency should be considered for particles smaller than 0.69 μm^61^. In this work, the size cut was set to 0.8 μm partly to be consistent with previous works. The WIBS was performed under forced trigger mode for 5 minutes every week. The signal level above which a particle could be attributed to emit fluorescence in those detection bands was determined by calculating the average and standard deviation in forced trigger mode. The thresholds for each fluorescence channel were set as the average fluorescence intensities plus 3-fold standard deviations. The threshold for every particle were linearly interpolated between two forced trigger measurement internals based on detection time. The 3-fold criterion was determined by examining the histogram of particle events in both FT mode and AC mode^62^.

To compare the results of the on-line and off-line fluorescent techniques, the total fluorescence intensity of the protein-like zone of EEM (excitation 275–285 nm/emission 330–400 nm) was compared with the total number counts of FL1 channel particles of WIBS. The result presented in Fig. 4 shows a good correlation between them which gives confidence to compare the results from these two methods. However, it should be noted that the data used here were confined to the first 12 samples for the overall 16 specimens. Because of the low concentrations of aerosols collected in the last 4 samples, the fluorescence intensity for offline measurement in this zone were below detection limit. Thus, they were eliminated from this comparison.

## Additional Information

**How to cite this article**: Yue, S. *et al*. Springtime precipitation effects on the abundance of fluorescent biological aerosol particles and HULIS in Beijing. *Sci. Rep.* **6**, 29618; doi: 10.1038/srep29618 (2016).

## Supplementary Material

Supplementary Information

## Figures and Tables

**Table 1:** Concentrations of organic and nitrogen components before, during and after the rain event.

Variables	Before 4/30 17:32–5/1 11:22	During 5/1 14:24–20:21	After 5/1 20:23–5/2 17:17
OC (μgC m^−3^)	13.7–20.6 (16.8)	37.8–41.6 (39.7)	11.4–25.9 (15.8)
WIOC (μgC m^−3^)	9.4–13.8 (11.5)	28.7–32.6 (30.7)	9.5–19.2 (13.6)
WSOC (μgC m^−3^)	4.1–6.8 (5.3)	9.0–9.0 (9.0)	1.3–6.7 (2.9)
WSTN (μgN m^−3^)	3.8–9.0 (6.7)	2.3–14.3 (8.3)	0.5–1.4 (0.9)

Data shown are minimum, maximum and average values during the corresponding periods.

## References

[b1] Després V. R. . Primary biological aerosol particles in the atmosphere: a review. Tellus B 64, 15598, doi: 10.3402/tellusb.v64i0.15598 (2012).

[b2] Jaenicke R. Abundance of cellular material and proteins in the atmosphere. Science (New York, NY) 308, 73, doi: 10.1126/science.1106335 (2005). 15802596

[b3] Graber E. R. & Rudich Y. Atmospheric HULIS: How humic-like are they? A comprehensive and critical review. Atmospheric Chemistry and Physics 6, 729–753, doi: 10.5194/acp-6-729-2006 (2006).

[b4] Feczko T. . Determination of water and alkaline extractable atmospheric humic‐like substances with the TU Vienna HULIS analyzer in samples from six background sites in Europe. Journal of Geophysical Research: Atmospheres (1984–2012) 112, D23S10, doi: 10.1029/2006JD008331 (2007).

[b5] Zheng G., He K., Duan F., Cheng Y. & Ma Y. Measurement of humic-like substances in aerosols: A review. Environmental Pollution 181, 301–314, doi: 10.1016/j.envpol.2013.05.055 (2013). 23830737

[b6] Andreae M. O. & Rosenfeld D. Aerosol–cloud–precipitation interactions. Part 1. The nature and sources of cloud-active aerosols. Earth-Science Reviews 8, 13–41, doi: 10.1016/j.earscirev.2008.03.001 (2008).

[b7] Kulmala M. . General overview: European Integrated project on Aerosol Cloud Climate and Air Quality interactions (EUCAARI) – integrating aerosol research from nano to global scales. Atmospheric Chemistry and Physics 11, 13061–13143, doi: 10.5194/acp-11-13061-2011 (2011).

[b8] Fuzzi S. . Particulate matter, air quality and climate: lessons learned and future needs. Atmospheric Chemistry and Physics 15, 8217–8299, doi: 10.5194/acp-15-8217-2015 (2015).

[b9] Pöschl U. . Rainforest aerosols as biogenic nuclei of clouds and precipitation in the Amazon. Science (New York, NY) 329, 1513–1516, doi: 10.1126/science.1191056 (2010). 20847268

[b10] Pöhlker C. . Biogenic potassium salt particles as seeds for secondary organic aerosol in the Amazon. Science (New York, NY) 337, 1075–1078, doi: 10.1126/science.1223264 (2012). 22936773

[b11] DeLeon-Rodriguez N. . Microbiome of the upper troposphere: species composition and prevalence, effects of tropical storms, and atmospheric implications. Proceedings of the National Academy of Sciences of the United States of America 110, 2575–2580, doi: 10.1073/pnas.1212089110 (2013). 23359712 PMC3574924

[b12] Steiner A. L. . Pollen as atmospheric cloud condensation nuclei. Geophysical Research Letters 42, 3596–3602, doi: 10.1002/2015GL064060 (2015).

[b13] O’Sullivan D. . The relevance of nanoscale biological fragments for ice nucleation in clouds. Scientific Reports 5, 8082, doi: 10.1038/srep08082 (2015). 25626414 PMC4308702

[b14] Maki L. R., Galyan E. L., Chang-Chien M. M. & Caldwell D. R. Ice nucleation induced by pseudomonas syringae. Applied microbiology 28, 456–459 (1974). 4371331 10.1128/am.28.3.456-459.1974 PMC186742

[b15] Andreae M. O. Atmospheric Aerosols: Biogeochemical Sources and Role in Atmospheric Chemistry. Science 276, 1052–1058, doi: 10.1126/science.276.5315.1052 (1997).

[b16] Möhler O., DeMott P., Vali G. & Levin Z. Microbiology and atmospheric processes: the role of biological particles in cloud physics. Biogeosciences 4, 1059–1071, doi: 10.5194/bg-4-1059-2007 (2007).

[b17] Amato P. . Survival and ice nucleation activity of bacteria as aerosols in a cloud simulation chamber. Atmospheric Chemistry and Physics Discussions 15, 6455–6465, doi: 10.5194/acpd-15-4055-2015 (2015).

[b18] Hiranuma N. . Ice nucleation by cellulose and its potential contribution to ice formation in clouds. Nature Geoscience 8, 273–277, doi: 10.1038/ngeo2374 (2015).

[b19] Vaïtilingom M. . Potential impact of microbial activity on the oxidant capacity and organic carbon budget in clouds. Proceedings of the National Academy of Sciences of the United States of America 110, 559–564, doi: 10.1073/pnas.1205743110 (2013). 23263871 PMC3545818

[b20] Mortazavi R., Attiya S. & Ariya P. A. Arctic microbial and next-generation sequencing approach for bacteria in snow and frost flowers: selected identification, abundance and freezing nucleation. Atmospheric Chemistry and Physics 15, 6183–6204, doi: 10.5194/acp-15-6183-2015 (2015).

[b21] Taraniuk I., Graber E. R., Kostinski A. & Rudich Y. Surfactant properties of atmospheric and model humic‐like substances (HULIS). Geophysical Research Letters 34, L16807, doi: 10.1029/2007GL029576 (2007).

[b22] Limbeck A., Kulmala M. & Puxbaum H. Secondary organic aerosol formation in the atmosphere via heterogeneous reaction of gaseous isoprene on acidic particles. Geophysical Research Letters 30, 1996, doi: 10.1029/2003GL017738 (2003).

[b23] Laskin A., Laskin J. & Nizkorodov S. A. Chemistry of Atmospheric Brown Carbon. Chemical reviews 115, 4335–4382, doi: 10.1021/cr5006167 (2015). 25716026

[b24] Pöschl U. & Shiraiwa M. Multiphase chemistry at the atmosphere-biosphere interface influencing climate and public health in the anthropocene. Chemical reviews 115, 4440–4475, doi: 10.1021/cr500487s (2015). 25856774

[b25] Chen P.-S. . Airborne Transmission of Melioidosis to Humans from Environmental Aerosols Contaminated with B. pseudomallei. PLOS Neglected Tropical Diseases 9, e0003834, doi: 10.1371/journal.pntd.0003834 (2015). 26061639 PMC4462588

[b26] Lin P. & Yu J. Generation of Reactive Oxygen Species Mediated by Humic-like Substances in Atmospheric Aerosols. Environmental Science & Technology 45, 10362–10368, doi: 10.1021/es2028229 (2011). 22044074

[b27] Fang Z. . Culturable airborne fungi in outdoor environments in Beijing, China. Science of The Total Environment 350, 47–58, doi: 10.1016/j.scitotenv.2005.01.032 (2005). 16227072

[b28] Dou J., Lin P., Kuang B.-Y. & Yu J. Reactive Oxygen Species Production Mediated by Humic-like Substances in Atmospheric Aerosols: Enhancement Effects by Pyridine, Imidazole, and Their Derivatives. Environmental Science & Technology 49, 6457–6465, doi: 10.1021/es5059378 (2015). 25961507

[b29] Hirst J. M. & Stedman O. J. DRY LIBERATION OF FUNGUS SPORES BY RAINDROPS. Journal of general microbiology 33, 335–344, doi: 10.1099/00221287-33-2-335 (1963). 14121209

[b30] Prenni A. J. . The impact of rain on ice nuclei populations at a forested site in Colorado. Geophysical Research Letters 40, 227–231, doi: 10.1029/2012gl053953 (2013).

[b31] Huffman J. A. . High concentrations of biological aerosol particles and ice nuclei during and after rain. Atmospheric Chemistry and Physics 13, 6151–6164, doi: 10.5194/acp-13-6151-2013 (2013).

[b32] Hirst J. M. Changes in atmospheric spore content: Diurnal periodicity and the effects of weather. Transactions of the British Mycological Society 36, 375–393, doi: 10.1016/S0007-1536(53)80034-3 (1953).

[b33] Allitt U. Airborne fungal spores and the thunderstorm of 24 June 1994. Aerobiologia 16, 397–406, doi: 10.1023/A:1026503500730 (2000).

[b34] Zhang Z. . Significant influence of fungi on coarse carbonaceous and potassium aerosols in a tropical rainforest. Environmental Research Letters 10, 034015, doi: 10.1088/1748-9326/10/3/034015 (2015).

[b35] Fowler D. . Atmospheric composition change: Ecosystems–Atmosphere interactions. Atmospheric Environment 43, 5193–5267, doi: 10.1016/j.atmosenv.2009.07.068 (2009).

[b36] Kaye . Single particle multichannel bio-aerosol fluorescence sensor. Optics Express 13, 3583–3593, doi: 10.1364/OPEX.13.003583 (2005). 19495264

[b37] Gabey A. M. . Measurements and comparison of primary biological aerosol above and below a tropical forest canopy using a dual channel fluorescence spectrometer. Atmospheric Chemistry and Physics 10, 4453–4466, doi: 10.5194/acp-10-4453-2010 (2010).

[b38] Pöhlker C., Huffman J. A. & Pöschl U. Autofluorescence of atmospheric bioaerosols – fluorescent biomolecules and potential interferences. Atmospheric Measurement Techniques 5, 37–71, doi: 10.5194/amt-5-37-2012 (2012).

[b39] Bauer H. . The contribution of bacteria and fungal spores to the organic carbon content of cloud water, precipitation and aerosols. Atmospheric Research 64, 109–119, doi: 10.1016/S0169-8095(02)00084-4 (2002).

[b40] Duan F., Liu X., Yu T. & Cachier H. Identification and estimate of biomass burning contribution to the urban aerosol organic carbon concentrations in Beijing. Atmospheric Environment 38, 1275–1282, doi: 10.1016/j.atmosenv.2003.11.037 (2004).

[b41] Elbert W., Taylor P. E., Andreae M. O. & Pöschl U. Contribution of fungi to primary biogenic aerosols in the atmosphere: wet and dry discharged spores, carbohydrates, and inorganic ions. Atmospheric Chemistry and Physics 7, 4569–4588, doi: 10.5194/acp-7-4569-2007 (2007).

[b42] Havers N., Burba P., Lambert J. & Klockow D. Spectroscopic Characterization of Humic-Like Substances in Airborne Particulate Matter. Journal of Atmospheric Chemistry 29, 45–54, doi: 10.1023/A:1005875225800 (1998).

[b43] Fu P. . Fluorescent water-soluble organic aerosols in the High Arctic atmosphere. Scientific Reports 5, 9845, doi: 10.1038/srep09845 (2015). 25920042 PMC4412076

[b44] Lindemann J., Constantinidou H. A., Barchet W. R. & Upper C. D. Plants as sources of airborne bacteria, including ice nucleation-active bacteria. Applied and environmental microbiology 44, 1059–1063 (1982). 16346129 10.1128/aem.44.5.1059-1063.1982 PMC242148

[b45] Joung Y. & Buie C. R. Aerosol generation by raindrop impact on soil. Nature Communications 6, 6083, doi: 10.1038/ncomms7083 (2015). 25586153

[b46] Butterworth J. & McCartney H. A. The dispersal of bacteria from leaf surfaces by water splash. Journal of Applied Bacteriology 71, 484–496, doi: 10.1111/j.1365-2672.1991.tb03822.x (1991).

[b47] Leach C. M. An Electrostatic Theory to Explain Violent Spore Liberation by Drechslera turcica and Other Fungi. Mycologia 68, 63–86, doi: 10.2307/3758898 (1976). 945457

[b48] Fu P. Q. . Aircraft measurements of polar organic tracer compounds in tropospheric particles (PM10) over central China. Atmospheric Chemistry and Physics 14, 4185–4199, doi: 10.5194/acp-14-4185-2014 (2014).

[b49] Rathnayake C. M. . Urban enhancement of PM10 bioaerosol tracers relative to background locations in the Midwestern United States. Journal of Geophysical Research: Atmospheres, doi: 10.1002/2015JD024538 (2016). PMC5034947 27672535

[b50] Fu P. . Molecular markers of biomass burning, fungal spores and biogenic SOA in the Taklimakan desert aerosols. Atmospheric Environment 130, 64–73, doi: 10.1016/j.atmosenv.2015.10.087 (2016).

[b51] Miyazaki Y., Fu P., Ono K., Tachibana E. & Kawamura K. Seasonal cycles of water‐soluble organic nitrogen aerosols in a deciduous broadleaf forest in northern Japan. Journal of Geophysical Research: Atmospheres 119, 1440–1454, doi: 10.1002/2013JD020713 (2014).

[b52] Zhu C., Kawamura K. & Kunwar B. Organic tracers of primary biological aerosol particles at subtropical Okinawa Island in the western North Pacific Rim. Journal of Geophysical Research: Atmospheres 120, 5504–5523, doi: 10.1002/2015JD023611 (2015).

[b53] Graham B. . Organic compounds present in the natural Amazonian aerosol: Characterization by gas chromatography–mass spectrometry. Journal of Geophysical Research: Atmospheres (1984–2012) 108, 4766, doi: 10.1029/2003JD003990 (2003).

[b54] McKnight D. M. . Spectrofluorometric characterization of dissolved organic matter for indication of precursor organic material and aromaticity. Limnology and Oceanography 46, 38–48, doi: 10.4319/lo.2001.46.1.0038 (2001).

[b55] Huguet A. . Properties of fluorescent dissolved organic matter in the Gironde Estuary. Organic Geochemistry 40, 706–719, doi: 10.1016/j.orggeochem.2009.03.002 (2009).

[b56] Fasching C., Behounek B., Singer G. A. & Battin T. J. Microbial degradation of terrigenous dissolved organic matter and potential consequences for carbon cycling in brown-water streams. Scientific Reports 4, 4981, doi: 10.1038/srep04981 (2014). 24828296 PMC4021337

[b57] Birdwell J. E. & Valsaraj K. T. Characterization of dissolved organic matter in fogwater by excitation–emission matrix fluorescence spectroscopy. Atmospheric Environment 44, 3246–3253, doi: 10.1016/j.atmosenv.2010.05.055 (2010).

[b58] Liu C. & Zipser E. J. The global distribution of largest, deepest, and most intense precipitation systems. Geophysical Research Letters 42, 3591–3595, doi: 10.1002/2015GL063776 (2015).

[b59] Coble P. G. Marine Optical Biogeochemistry: The Chemistry of Ocean Color. Chemical Reviews 107, 402–418, doi: 10.1021/cr050350+ (2007). 17256912

[b60] O’Connor D. J., Healy D. A. & Sodeau J. R. A 1-month online monitoring campaign of ambient fungal spore concentrations in the harbour region of Cork, Ireland. Aerobiologia 31, 295–314, doi: 10.1007/s10453-015-9365-7 (2015).

[b61] Healy D. A., O’Connor D. J. & Sodeau J. R. Measurement of the particle counting efficiency of the “Waveband Integrated Bioaerosol Sensor” model number 4 (WIBS-4). Journal of Aerosol Science 47, 94–99, doi: 10.1016/j.jaerosci.2012.01.003 (2012).

[b62] Perring A. E. . Airborne observations of regional variation in fluorescent aerosol across the United States. Journal of Geophysical Research: Atmospheres 120, 1153–1170, doi: 10.1002/2014JD022495 (2015).

