# Supplementary material for: Springtime precipitation effects on the abundance of fluorescent biological aerosol particles and HULIS in Beijing
Source: Sci Rep. 2016 Jul 29;6:29618. doi: 10.1038/srep29618 (PMC4965869; doi:10.1038/srep29618)
Supplement: Supplementary Information [file srep29618-s1.pdf]

Supplementary Information

Springtime precipitation effects on the abundance of fluorescent biological aerosol particles and HULIS in Beijing

Siyao Yue [1,2], Hong Ren [1,2], Songyun Fan [1], Yele Sun [1,3], Zifa Wang [1,3], and Pingqing Fu [1,3*]

[1] State Key Laboratory of Atmospheric Boundary Layer Physics and Atmospheric Chemistry, Institute of Atmospheric Physics, Chinese Academy of Sciences, Beijing 100029, China

[2] College of Earth Sciences, University of Chinese Academy of Sciences, Beijing 100049, China

[3] Center for Excellence in Urban Atmospheric Environment, Institute of Urban Environment, Chinese Academy of Sciences, Xiamen 361021, China

*Corresponding author e-mail: fupingqing@mail.iap.ac.cn

This file contains seven pages with five figures (Figures S1-S5) and one table (Table S1).

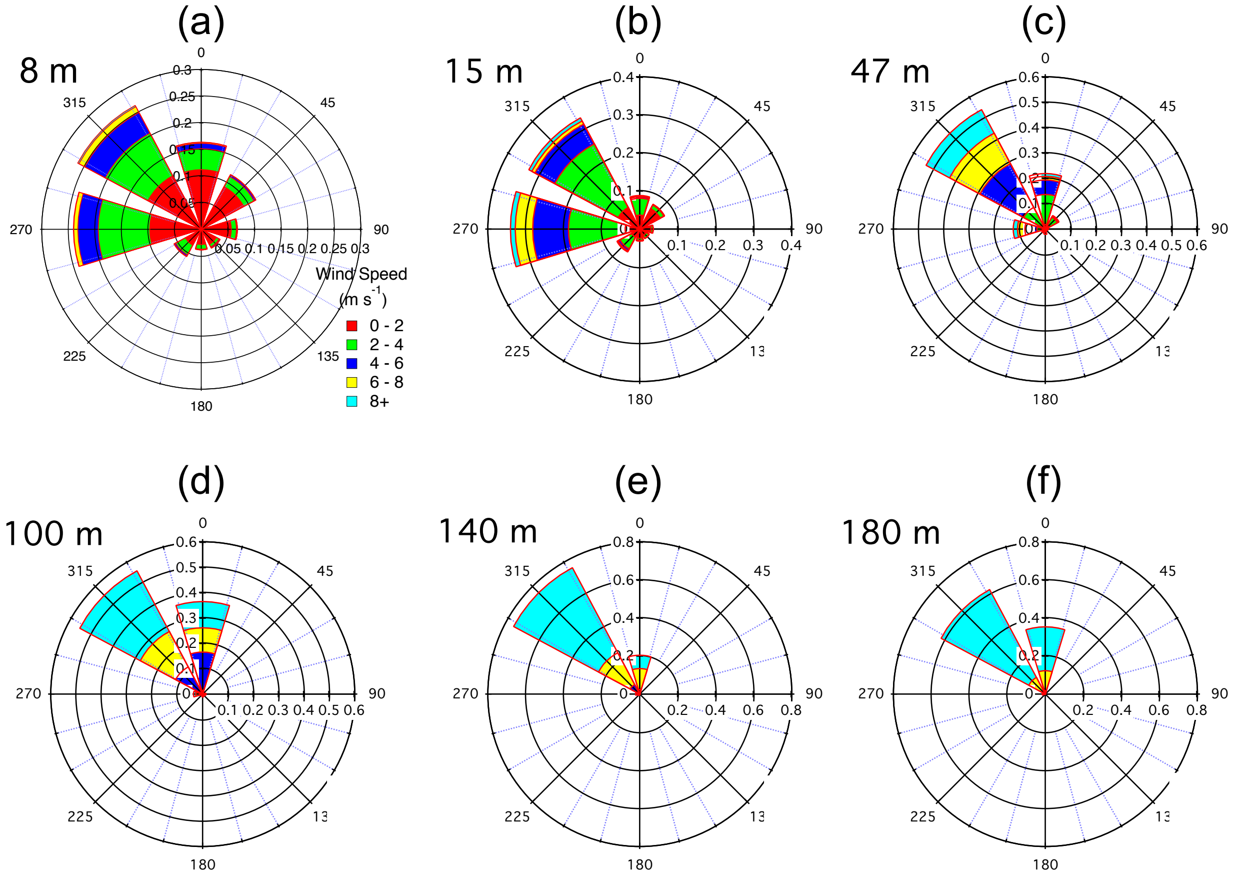

Figure S1. Rose plot of the wind in the time interval May 1, 16:40 - 19:40 for different layers measured on the tower. The rose plot's radius is the probability that a velocity value is within the given direction bin.

Figure S2 and Figure S3 show that no significant correlations between WIBS FL2, FL3 channels and off-line EEM HULIS fluorescence zones. Though the HULIS detected here are constrained to water-soluble proportions, it is, to some extent, reasonable to eliminate possible interference of HULIS on WIBS yields.

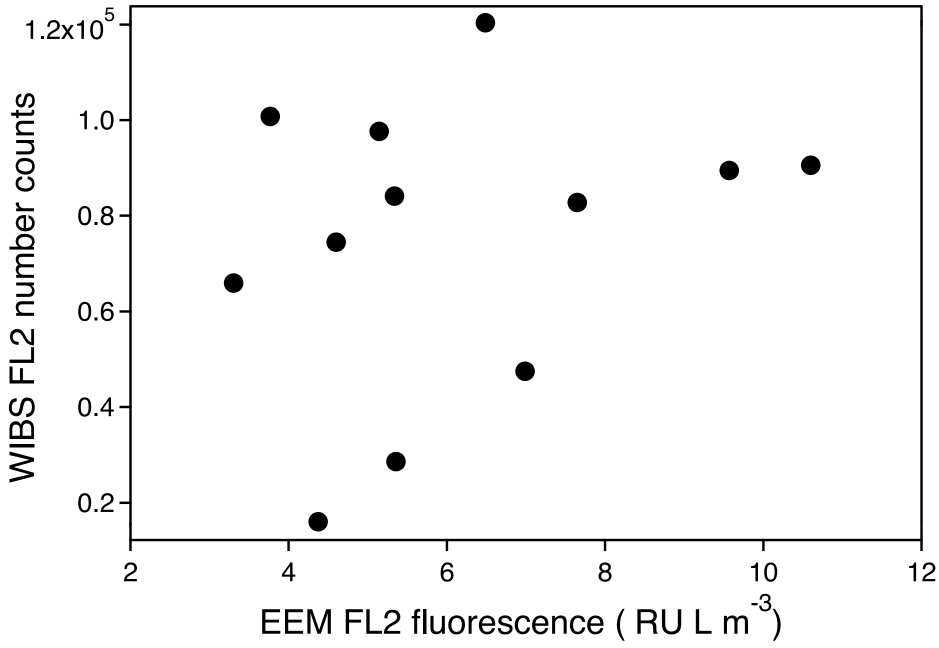

Figure S2. Scatter plot of number count of particles from WIBS FL2 fluorescence channel and EEM fluorescence intensity integrated in the corresponding excitation-emission zone (the rectangle defined by Excitation/Emission: 275-285 nm/420-524 nm).

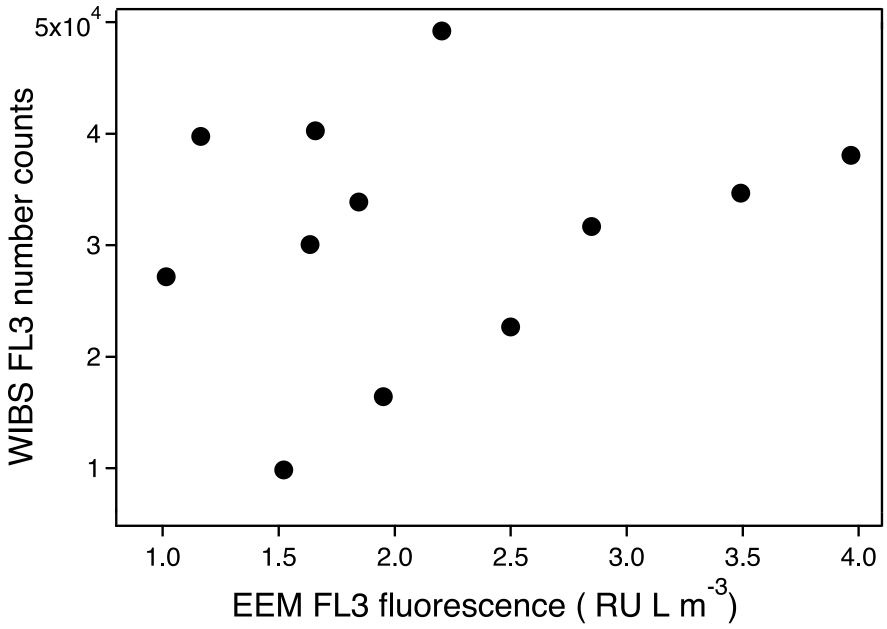

Figure S3. Scatter plot of number count of particles from WIBS FL3 fluorescence channel and EEM fluorescence intensity integrated in the corresponding excitation-emission zone (the rectangle defined by excitation/emission: 365-375 nm/420-524 nm).

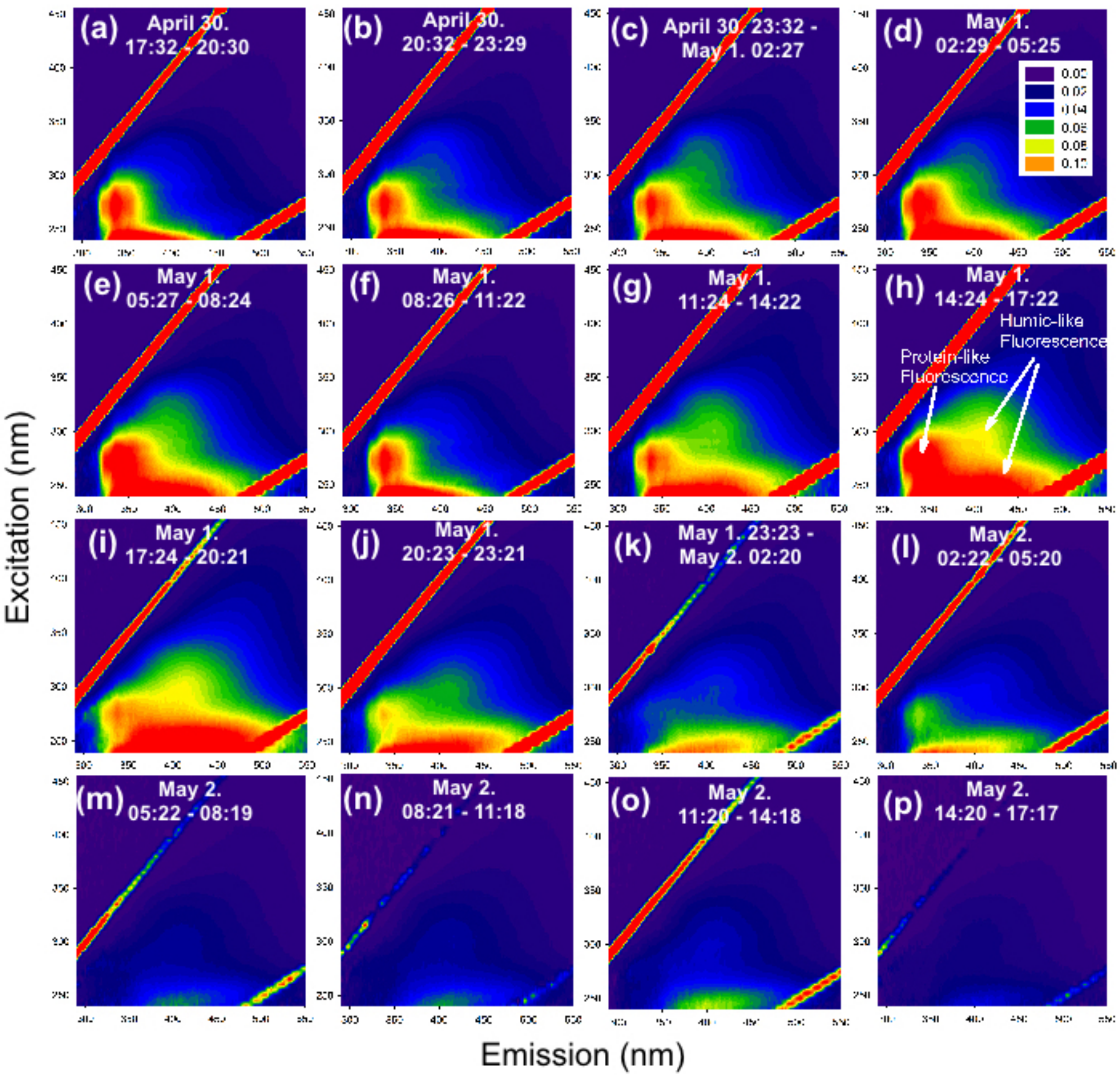

Figure S4. Time series of fluorescence spectra (EEM) of water-soluble organic carbon (WSOC) (in RU L m^{-3}) before, during and after the precipitation.

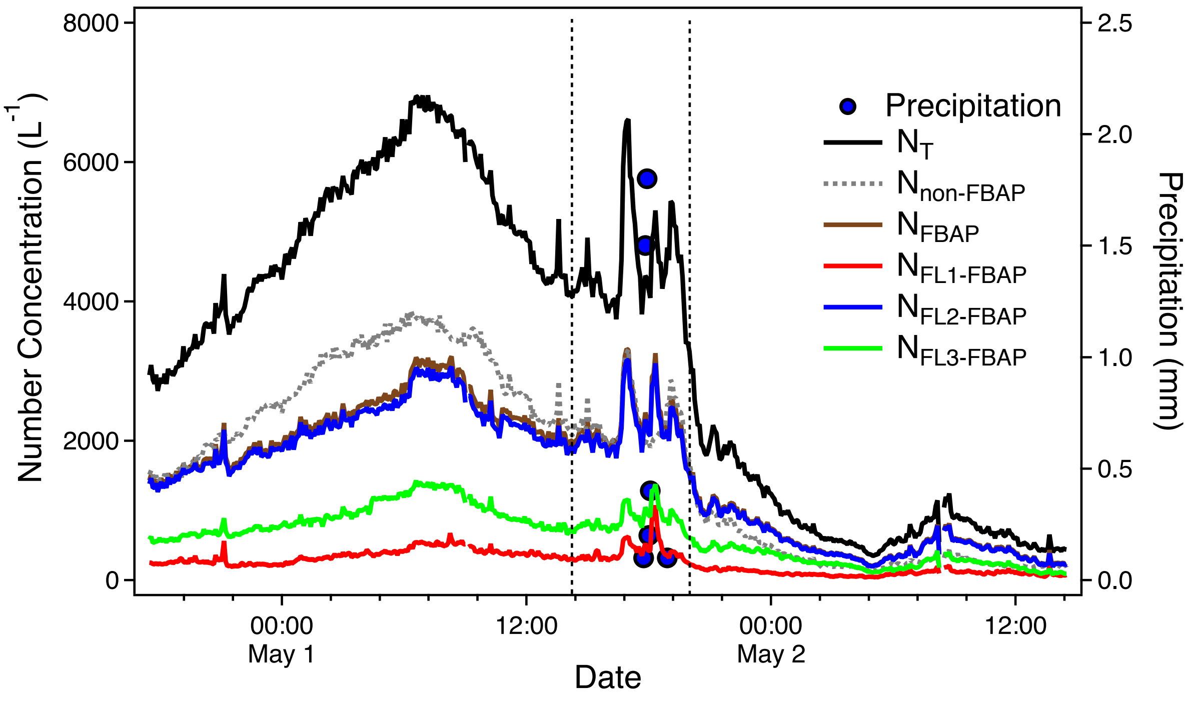

Figure S5. Extended temporal variations of aerosol number concentrations (for particles > 0.8 *μm*, optically scattering diameter) observed by WIBS and rainfall rate. Variables presented are number concentrations of total particles (N_T), fluorescent biological aerosol particles (N_{FBAP}), three different channels of fluorescent biological aerosol particles ($N_{FL1-FBAP}$, $N_{FL2-FBAP}$ and $N_{FL3-FBAP}$) and non-fluorescent biological aerosol particles ($N_{non-FBAP}$). Five-minute accumulated rainfall rate is shown by blue dots. The time span between the two dotted lines is discussed in the main article (Figure 1).

Table S1. The number concentrations of FBAP, FL1-FBAP, FL2-FBAP, FL3-FBAP and non-FBAP for 5 size modes (0.9 *μm*, 1.2 *μm*, 2.2 *μm*, 2.8 *μm* and total particles larger than 3 *μm*) in the five time periods.

Size modes (μm)	Before Rain (L^{-1})	Rainfall (L^{-1})			After Rain (L^{-1})
	15:40 – 16:40	16:40 – 17:40	17:40 – 18:40	18:40 – 19:40	19:40 – 20:40
non-FBAP					
0.9	343.5	353.2	271.1	317.8	211.0
1.2	208.3	257.1	187.8	223.1	149.3
2.2	75.4	161.1	121.1	163.8	100.7
2.8	10.2	29.9	23.8	37.2	24.1
> 3	11.5	29.1	23.7	40.7	22.8
FBAP					
0.9	209.4	187.0	261.4	194.3	149.3
1.2	162.3	153.7	184.9	134.4	102.2
2.2	125.5	223.7	180.3	178.5	122.1
2.8	48.9	131.3	91.7	112.3	62.6
> 3	88.4	292.3	185.2	252.9	134.0
FL1-FBAP					
0.9	30.2	25.1	59.4	19.5	13.8
1.2	23.9	23.5	52.1	19.5	13.3
2.2	16.9	34.6	34.0	22.8	15.9
2.8	10.7	28.5	21.2	21.7	12.5
> 3	27.4	107.0	61.6	84.0	44.8
FL2-FBAP					
0.9	197.3	174.0	248.3	184.2	143.0
1.2	153.7	145.3	177.1	126.2	96.4
2.2	121.0	213.5	173.3	172.0	117.0
2.8	47.8	128.4	89.5	108.7	60.8
> 3	88.2	288.7	181.4	249.1	132.2
FL3-FBAP					
0.9	58.5	65.6	107.4	87.0	63.4
1.2	50.1	48.6	80.0	52.3	39.2
2.2	54.4	64.5	65.6	51.6	37.1
2.8	28.0	58.5	46.3	47.5	28.4
> 3	59.9	179.4	118.1	145.7	78.9
